# Effects of permissive hypercapnia on intraoperative cerebral oxygenation and early postoperative cognitive function in elderly patients undergoing laparoscopic surgery

**DOI:** 10.3389/fmed.2025.1575412

**Published:** 2025-10-09

**Authors:** Huixian Chen, Yingqun Yu, Jiang Huo, Guijin Dou, Qianqian Zhao, Shuang Zhao, Fuqi Zhao

**Affiliations:** Department of Anesthesiology, The Fifth Medical Center, Chinese PLA General Hospital, Beijing, China

**Keywords:** permissive hypercapnia, laparoscopic surgery, elderly patients, cerebral oxygenation, early cognitive function

## Abstract

**Background:**

This study examines the effect of permissive hypercapnia (PH) on cerebral oxygenation (rSO_2_) and early postoperative cognitive function in elderly patients (EP) undergoing laparoscopic surgery (LS).

**Methods:**

This retrospective study analyzed data from elderly patients who underwent laparoscopic surgery with PH mechanical ventilation or conventional ventilation (CV) ventilation strategies between 2019 and 2024. The individuals were separated into two groups as stated by the intraoperative anesthesia method, and equal numbers in each group. A total of 550 patients were initially screened for eligibility, of whom 100 were excluded based on predefined criteria. The final analysis included 450 patients, with 225 receiving PH mechanical ventilation (PH group) and 225 receiving conventional ventilation (CV, control group). Postoperative cognitive dysfunction (POCD), postoperative regional cerebral oxygen saturation (rSO_2_), and cognitive function (measured by the Mini-Mental State Examination, MMSE) were compared involving the two groupings. Secondary outcomes included postoperative recovery time, bed rest time, hospital stay, postoperative complications, and intraoperative vital signs (blood pressure, heart rate, and arterial partial pressure of carbon dioxide).

**Results:**

Permissive hypercapnia was associated with significantly improved intraoperative cerebral oxygenation (rSO_2_) compared to conventional ventilation (mean difference 4.62%, 95% CI 3.81–5.43; *P* < 0.001), particularly following pneumoperitoneum establishment. MMSE scores demonstrated less pronounced decline at postoperative day 1 in the PH group (23.05 ± 0.23) versus controls (20.67 ± 1.63; *P* < 0.001), with recovery to baseline by day 14 (24.87 ± 1.23 vs. 23.91 ± 1.51; *P* = 0.012), with MMSE scores higher than those in the CV group (*P* < 0.05). Secondary outcomes, including recovery time and vital signs, did not differ significantly between groups (*P* > 0.05).

**Conclusion:**

Permissive hypercapnia significantly improves rSO_2_ and was associated with smaller early declines in MMSE scores, suggesting a potential benefit on global cognition. These findings are exploratory and should be confirmed using comprehensive neuropsychological batteries.

## Introduction

Laparoscopic surgery (LS) is widely used in various surgical operations due to its advantages such as less trauma, faster recovery, and fewer complications, especially for elderly patients (EP). However, EP are particularly sensitive to the choice of anesthesia strategy due to the presence of multiple chronic diseases and organ function degeneration during perioperative management. During LS, the establishment of pneumoperitoneum can easily lead to increased intra-abdominal pressure, which in turn affects respiratory function, hemodynamics and cerebral oxygenation status, which may bring additional risks to EP who already have cerebral insufficiency or cerebral function degeneration (1, 2).

Permissive hypercapnia (PH), as an anesthetic management strategy, has been shown to improve systemic circulation, enhance cerebral blood flow (CBF), and maintain cerebral oxygenation by tolerating mild carbon dioxide accumulation (3, 4). In addition, the cerebral vasodilatation (CVD) induced by hypercapnia helps prevent intraoperative hypoxemia. However, in EP, cerebrovascular regulation is often impaired, making them less tolerant to functions in carbon dioxide concentration (5). As a result, PH may have a dual impact on cerebral oxygenation and postoperative cognitive function (PCF) in this population (6). Research has shown that inadequate cerebral oxygenation is strongly associated with postoperative cognitive dysfunction (POCD), a common and serious complication in EP that can significantly affect their quality of life and prognosis (7, 8). Therefore, it is clinically important to explore the potential benefits of PH in LS, especially its effects on cerebral oxygenation and early PCF in EP. Properly controlling intraoperative carbon dioxide levels may help reduce the incidence of early POCD in these individuals, thus enhancing both surgical safety and overall outcomes. Despite the promising theoretical basis, current research on the clinical application of PH in EP undergoing LS is limited, especially regarding its effect on cerebral oxygenation and cognitive function. This study aimed to evaluate the effects of PH on intraoperative cerebral oxygenation and to explore its potential association with early postoperative global cognitive changes, assessed using MMSE, in elderly patients undergoing laparoscopic surgery. The findings could provide a scientific foundation for optimizing perioperative management in this patient population. While this study focuses on cerebral oxygenation and exploratory global cognitive changes, we recognize that evaluating patients’ quality of life (QoL) over a longer follow-up period, such as 6 months, would provide a more comprehensive understanding of the clinical impact of permissive hypercapnia.

## Materials and methods

### Research flow chart

**FIGURE 1:**
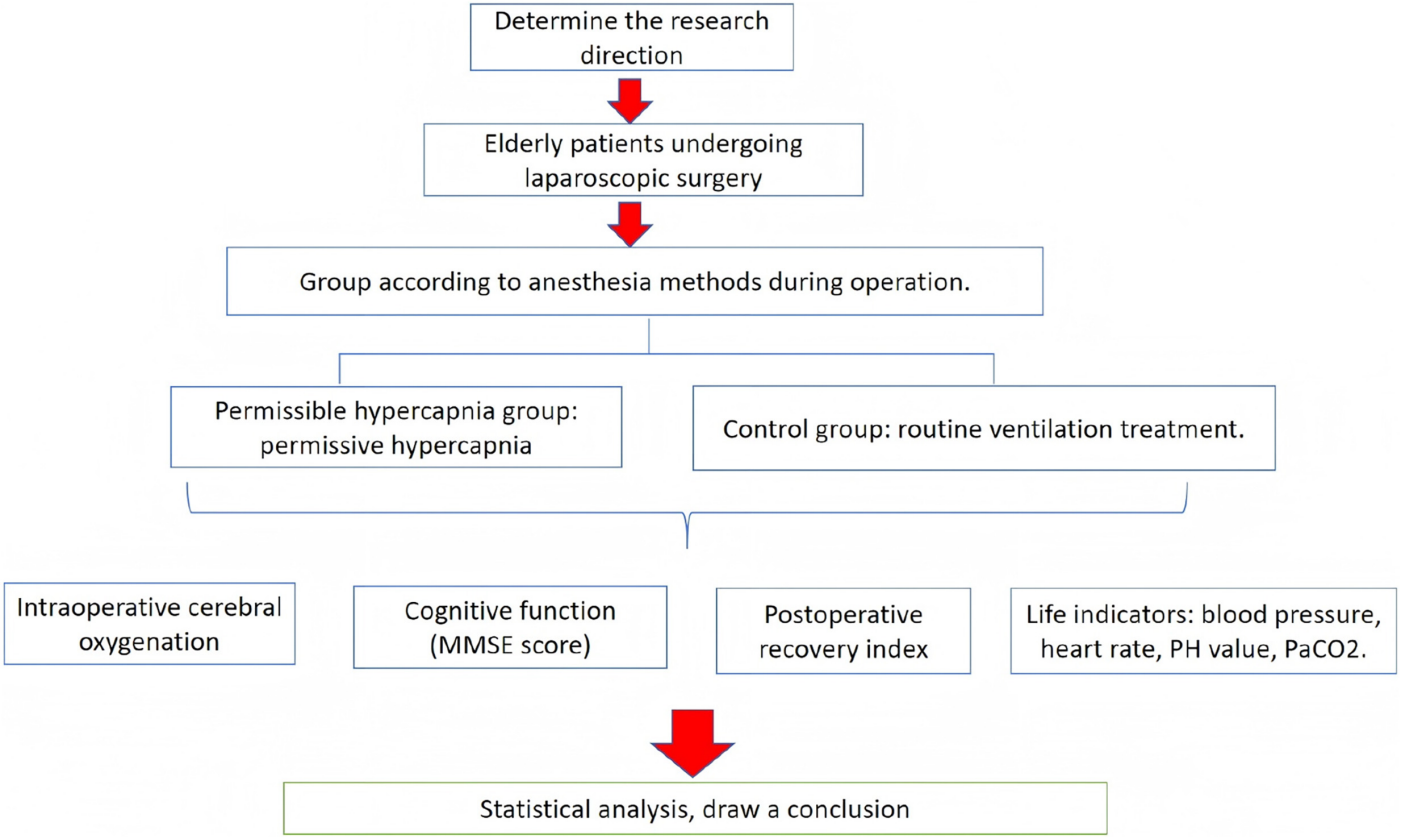
Research flow chart.

Figure 1 show the flow chart of the research.

### Study subjects

This retrospective study analyzed data from elderly patients who underwent laparoscopic surgery between March 2019 and March 2024. Patients were stratified into permissive hypercapnia (PH) or conventional ventilation (CV) groups based on intraoperative ventilation parameters documented in anesthesia records. The sum of 450 patients were included in the study, consisting of 225 individuals who received PH mechanical ventilation (PH group) and 225 patients who received conventional ventilation (CV, control group). Postoperative MMSE assessments at days 1, 7, 14, and 30 were routinely performed for all elderly surgical patients as part of standard cognitive monitoring.

Exposure definition and grouping. Patients were retrospectively classified as PH or CV based on documented intraoperative ventilation targets and arterial blood gas (ABG) values in the anesthesia record. PH was defined as intended PaCO_2_ target 45–65 mmHg with tidal volume 6–8 mL/kg predicted body weight and maintenance of PaCO_2_ ≥ 45 mmHg for ≥ 50% of intraoperative ABG timepoints (time-weighted when multiple ABGs were available). CV was defined as intended PaCO_2_ 35–45 mmHg with tidal volume 8–12 mL/kg actual body weight and maintenance of PaCO_2_ 35–45 mmHg for ≥ 50% of timepoints. When parameters varied, the time-weighted PaCO_2_/ventilator settings determined exposure. Patients with < 2 intraoperative ABGs were classified using the prespecified target documented by the anesthesiologist plus end-tidal CO_2_ trend notes.

Clinical rationale for ventilation choice. As this was a retrospective study, ventilation strategy was chosen at the discretion of the attending anesthesiologist. Factors commonly considered included anticipated pneumoperitoneum duration, baseline pulmonary reserve (e.g., mild preoperative hypoventilation/CO_2_ retention), hemodynamic stability, body habitus, and surgeon positioning/insufflation pressures. We therefore recognize the potential for non-random, preference-based allocation.

Cross-overs. If a case transitioned between strategies, we used an as-treated approach (time-weighted exposure); a per-protocol sensitivity analysis re-classified patients to the strategy maintained for ≥ 70% of intraoperative time.

Data source. Ventilator settings (VT, RR, FiO_2_), end-tidal CO_2_, and ABG values were abstracted from the anesthesia information management system and nursing flowsheets by two reviewers; discrepancies were resolved by consensus.

Inclusion criteria: (1) Elective surgery with clear indications for the procedure; (2) EP aged 65 years or older; (3) Patients with normal cognitive function, as assessed by the Mini-Mental State Examination (MMSE) (9) before surgery; (4) Complete clinical data available and voluntary signing of the informed consent form.

Exclusion criteria: (1) Long-term use of medications that affect mental state; (2) Comorbid mental and psychological disorders resulting in impaired cognitive function; (3) A history of craniocerebral trauma; (4) Low educational level, defined as fewer than 6 years of formal education or inability to complete basic cognitive assessments, or lack of basic cognitive ability (e.g., illiterate); (5) Presence of language or visual impairments that hinder normal communication and cooperation during the examination; (6) Inability to communicate fluently in Mandarin, leading to potential communication errors; (7) Allergic to anesthetic drugs or related substances used during the perioperative period; (8) Early discharged due to disputes arising during the diagnosis and treatment process after admission.

### Sample size determination

The sample size was calculated *a priori* using PS Power and Sample Size software (version 3.1.2). Based on preliminary data from 50 patients showing a 3.0-point mean difference in postoperative MMSE scores between ventilation strategies (SD = 5.5 points), with Type I error (α) = 0.05 (two-tailed) and power (1–β) = 80%, the calculation indicated a minimum requirement of 198 patients per group. To account for potential 15% attrition rate (lost to follow-up, incomplete data) and covariate adjustment in final analyses, we enrolled 225 patients per group (total *N* = 450). *Post hoc* power analysis using observed effects confirmed 82.3% power to detect the primary outcome difference (MMSE at postoperative day 7), exceeding the conventional 80% power threshold and validating sample adequacy.

### Ethical approval

The current study was approved by the Ethics Committee of the Fifth medical center of Chinese PLA General Hospital (approval number FCH202407171). Due to the retrospective design and use of anonymized data, the requirement for written informed consent was waived by the Ethics Committee.

### Treatment

Prior to study inclusion, 100 patients were excluded for the following reasons: long-term psychoactive medication use (*n* = 22), pre-existing cognitive impairment (*n* = 18), language/educational barriers preventing Mini-Mental State Examination (MMSE) completion (*n* = 25), and early discharge or incomplete data collection (*n* = 35). The remaining 450 patients included in this study underwent laparoscopic surgery (LS) with intubation anesthesia. For the conventional ventilation (CV) group, standardized parameters were maintained: tidal volume (VT) 8–12 mL/kg, respiratory rate (RR) 12–14 breaths/min, inspiratory-to-expiratory ratio (I:E) 1:2, with arterial carbon dioxide partial pressure [p(CO_2_)] kept at 35–45 mmHg. Ventilation parameters were adjusted as needed based on blood gas analysis results. At our institution, permissive hypercapnia (target PaCO_2_ 45–65 mmHg) has been an approved ventilation strategy for laparoscopic surgery in elderly patients since 2018, while conventional normocapnic ventilation remained the default approach. The ventilation parameters of the PH mechanical ventilation group were as follows: VT6–8 mL/kg, RR12–14 times/min, respiratory ratio 1:2, maintaining p(CO_2_) 45–65 mmHg, pH value > 7.2, and adjusting ventilation indicators according to blood gas analysis results. Our selection of a higher PaCO_2_ threshold (45–65 vs. 45–55 mmHg in prior studies) was based on three key considerations: First, elderly patients often exhibit reduced cerebrovascular CO_2_ reactivity, requiring slightly higher PaCO_2_ to achieve equivalent cerebral vasodilation. Second, preliminary data from our institution showed that rSO_2_ optimization in this population typically occurred at PaCO_2_ 50–60 mmHg without adverse effects. Third, we maintained strict safety boundaries (pH > 7.2, no respiratory acidosis) and real-time ABG monitoring to ensure physiological tolerability. This approach balanced neuroprotective benefits against potential risks in our vulnerable cohort. All patients received standardized FiO_2_ (50%–60%) throughout surgery, adjusted only if pulse oximetry (SpO_2_) fell below 94%. Preoperative hemoglobin (Hb) levels were measured routinely, with intraoperative Hb checks performed if blood loss exceeded 300 mL.

Arterial blood gas (ABG) analysis was performed at the following time points to measure PaCO_2_ and titrate ventilation: (1) preoperatively (baseline), (2) after endotracheal intubation, (3) 10 min after pneumoperitoneum establishment, (4) every 30 min during surgery during surgery, and (5) before extubation. Continuous end-tidal carbon dioxide (PETCO_2_) monitoring was used for real-time assessment, with ABG values confirming target PaCO_2_ ranges (45–65 mmHg for PH group; 35–45 mmHg for CV group). Ventilation parameters (tidal volume or respiratory rate) were adjusted if PaCO_2_ deviated from the target range.

The conventional ventilation (CV) group received tidal volumes of 8–12 mL/kg actual body weight, reflecting our institutional standards for non-ARDS patients during the study period (2019–2024). This approach was maintained with strict monitoring to ensure end-inspiratory plateau pressures remained < 30 cm H_2_O. The permissive hypercapnia (PH) group received lung-protective ventilation with lower tidal volumes (6–8 mL/kg predicted body weight), consistent with current protective strategies (Supplementary Table 1).

### Postoperative oxygen management

All patients were monitored in the post-anesthesia care unit (PACU) with continuous pulse oximetry. Supplemental oxygen (2 L/min via nasal cannula) was administered only if SpO2 fell below 94% for > 30 s. This threshold was maintained until rSO_2_ recording completion. Oxygenation status was documented at all assessment timepoints.

### Ventilation monitoring protocol

Ventilation was continuously monitored throughout surgery to ensure that target PaCO_2_ levels were maintained according to group allocation. Ventilation parameters were adjusted according to the following criteria:

- PH group: EtCO_2_ > 65 mmHg or PaCO_2_ > 65 mmHg (decrease tidal volume)
- CV group: EtCO_2_ > 45 mmHg or PaCO_2_ > 45 mmHg (increase respiratory rate)

During CO_2_ insufflation, EtCO_2_ increases > 10% from baseline triggered immediate ABG analysis. Arterial blood gas measurements were performed at all specified timepoints regardless of EtCO_2_ values to confirm PaCO_2_ levels.

Patients in both groups fasted for 12 h before surgery and did not take anticholinergic drugs and antiserotonin drugs for 30 min before surgery. Upon patient admission, a multifunctional anesthetic monitor was used to regularly check non-invasive blood pressure (BP), electrocardiogram (ECG), heart rate (HR), end-tidal carbon dioxide (PETCO_2_), body temperature (T), pulse oxygen saturation (SpO_2_), and the bispectral index (BIS). Peripheral venous access was also established, and rSO_2_ was continually recorded and tracked. Peripheral venous access was established, and standard monitoring protocols were followed during anesthesia induction and throughout the surgical procedure.

Intravenous administration of midazolam (0.04 mg/kg), sufentanil (0.4–0.6 μg/kg), and propofol (1.5–2.0 mg/kg) was performed. Rocuronium (0.6–0.9 mg/kg) was administered intravenously when the BIS value dropped to between 45 and 55. Endotracheal intubation was then performed once adequate muscle relaxation was confirmed, and mechanical ventilation was initiated.

Anesthesia was maintained using propofol (4–6 mg/kg⋅h) and remifentanil (0.25–0.50 μg/h), with BIS values controlled between 40 and 60. After the initiation of artificial pneumoperitoneum, the respiratory rate and tidal volume were adjusted as needed to ensure PaCO_2_ remained within the target range for each group.

No awakening agents were administered postoperatively. Patients were transferred to the post-anesthesia care unit (PACU) for monitoring during recovery.

### Observation indicators

#### Primary and secondary outcomes

The primary outcomes of this study were rSO_2_ levels and early postoperative global cognitive changes, assessed using the MMSE. The secondary outcomes included postoperative recovery time, ambulation time, hospital stay duration, incidence of postoperative complications, and intraoperative vital signs (blood pressure, heart rate, pH, and PaCO_2_).

#### Intraoperative cerebral oxygenation

Near infrared spectroscopy (NIRS) was used to monitor and evaluate the intraoperative changes in rSO_2_. The monitoring time points were before surgery (baseline), 10 min after the establishment of pneumoperitoneum, during surgery (halfway through the surgery), before surgery (when the surgery was about to end), and upon awakening after surgery. Continuous rSO_2_ monitoring was performed using the INVOS™ 5100C Cerebral Oximeter (Medtronic, United States). Two rSO_2_ sensors (Adult SomaSensor^®^ SAFB-SM) were symmetrically placed on the patient’s forehead, 1–2 cm above the eyebrow and aligned with the frontal cortex, following the manufacturer’s recommendations. The device uses near-infrared spectroscopy (NIRS) at 730 nm and 810 nm wavelengths to calculate rSO_2_ values, representing the mixed venous-weighted (70:30 venous-to-arterial ratio) oxygen saturation in the underlying cerebral tissue (predominantly the prefrontal cortex).

#### Ventilation and arterial blood gas (ABG) monitoring

Continuous end-tidal CO_2_ (EtCO_2_) monitoring was maintained throughout surgery using mainstream capnography (Datex-Ohmeda, Finland). The EtCO_2_–PaCO_2_ gradient was calculated at each ABG measurement point (baseline, post-intubation, post-pneumoperitoneum, every 30 min during surger, and pre-extubation). During CO_2_ insufflation, any EtCO_2_ increase of > 10% from baseline triggered immediate ABG analysis to confirm PaCO_2_ levels.

#### Jugular bulb catheterization and cerebral oxygen validation

After anesthesia induction, a retrograde catheter was inserted into the right internal jugular vein and advanced to the jugular bulb for intermittent blood sampling to measure jugular venous oxygen saturation (SjvO_2_) and arterial-jugular venous CO_2_ differences. This allowed validation of NIRS-derived rSO_2_ values and assessment of cerebral metabolic demand, per institutional protocols for prolonged laparoscopic procedures in elderly patients. Heparin was used to seal the catheter, with a collection rate of less than 2 mL/min in the jugular bulb.

#### Cognitive function

Postoperative MMSE assessments at days 1, 7, 14, and 30 were routinely performed for all elderly surgical patients as part of standard cognitive monitoring.

Postoperative cognitive dysfunction in both groups was evaluated using the Mini-Mental State Examination (MMSE), which consists of five sections with a total score of 30 points. Postoperative cognitive change was evaluated exploratorily using MMSE at baseline and follow-up. We did not attempt to POCD, which typically requires a comprehensive neuropsychological battery assessing multiple cognitive domains (8). Cognitive assessments at 7, 14, and 30 days postoperatively were conducted during scheduled outpatient follow-up visits or via structured telephone interviews by trained assessors blinded to group allocation. The MMSE consists of five sections with a total score of 30 points. MMSE-based POCD was defined as: (1) ≥ 1 SD decline from baseline in ≥ 2 cognitive domains, calculated using age/education-adjusted z-scores (ref 8), and (2) absolute post-operative score < 24. This dual threshold ensured both statistical and clinical significance. MMSE assessments were performed in-person by blinded staff during routine postoperative hospital visits, with all scores documented in electronic medical records. In this retrospective study, we used the MMSE because it is widely applied in clinical practice and available in our dataset. However, we acknowledge that MMSE is primarily a global cognition screening tool and has limited sensitivity for detecting subtle or domain-specific deficits, particularly in long-term memory, executive function, and attention. Quality of life (QoL) outcomes were not assessed in this study, which we recognize as a limitation and an area for future research. The specific scoring criteria are as follows:

Time orientation (5 points): The topic is questioned about the date, season, month, week, and year.

Location orientation (5 points): The topic is questioned about their province, city, location, floor, and room.

Immediate memory (3 points): The topic is questioned to repeat the names of three objects.

Attention and calculation (5 points): The topic is questioned to subtract 7 five times consecutively or to spell the word “world” in reverse order.

Delayed memory (3 points): The topic is questioned to recall the names of the three objects mentioned earlier.

Language and execution (9 points): This includes object naming (2 points), repeating phrases (1 point), following three-step instructions (3 points), copying pictures (1 point), auditory comprehension (1 point), and language expression (1 point).

Cognitive function was assessed based on the total score: normal was 27–30 points, mild cognitive impairment was 21–26 points, moderate was 10–20 points, and severe was 0–9 points. The assessment was conducted at the following time points: 1 day before surgery (baseline), 1, 7, 14 and 30 days after surgery.

#### Recovery indicators

Incidence of surgical complications, postoperative recovery time, postoperative ambulation time, and postoperative hospital stay days were statistically analyzed involving the two groupings of patients.

#### Vital indicators

Preoperative and intraoperative vital indexes, including systolic blood pressure, diastolic blood pressure, HR, PH and arterial partial pressure of carbon dioxide (PaCO_2_), were monitored.

### Statistical analysis

The statistical program SPSS24.0 was used. The measurement data underwent a normal distribution and variance homogeneity test prior to statistical analysis. They were all represented as ($\bar{x}$ ± s) and satisfied the criteria for a normal distribution or an approximation normal distribution. Repeated measurement variance analysis was used to examine the data from repeated measurements. The two categories were compared using the independent sample *t*-test, while comparisons within the group were compared using the paired *t*-test. *n* (%) was used to represent count data. *Post hoc* power analysis confirmed 82.3% power to detect the observed MMSE differences. Primary analyses were covariate-adjusted as specified above; effect sizes are presented as adjusted mean differences (with 95% CIs) for MMSE trajectories and adjusted odds ratios for MMSE-defined global cognitive change (exploratory). Sensitivity analyses used as-treated vs. per-protocol exposure and, when applicable, IPTW propensity weighting. *P* < 0.05 suggested that the difference was statistically significant according to the χ2 test.

### Propensity score analysis (sensitivity)

We estimated each patient’s propensity for PH using logistic regression including all covariates above plus interaction terms (age × ASA, COPD × operative time). We applied inverse probability of treatment weighting (IPTW) stabilized by the marginal treatment probability. Balance was assessed by standardized mean differences (SMD); SMD < 0.10 indicated adequate balance. IPTW-adjusted mixed models and logistic models were used to estimate treatment effects.

## Results

### Baseline data

Comparison of baseline data involving the two groupings showed no appreciable variations in gender, age, body mass index, proportion of first surgery, ethnicity, disease composition, educational background, payment method, marital status, and surgical grade (*P* > 0.05, Table 1). Comparison of baseline data between the two groups showed no significant differences in gender, age, BMI, first surgery proportion, comorbidities, and other baseline characteristics (*P* > 0.05).

**TABLE 1:** Baseline characteristics comparison ($\bar{x}$ ± s).

Characteristic	Permissive hypercapnia group (*n* = 225)	Normal group (*n* = 225)	*χ*^2^/t-value	*P*-value
Gender			0.036	0.850
Male	118 (55.24)	120 (53.33)	–	–
Female	107 (47.56)	105 (46.67)	–	–
Age (years)	68.21 ± 7.06	67.95 ± 8.11	0.363	0.717
Body mass index (kg/m^2^)	27.01 ± 2.65	26.84 ± 2.77	0.665	0.506
Initial surgery	198 (88.00)	187 (83.11)	2.176	0.140
Nationality			0.343	0.558
Chinese	220 (97.78)	218 (96.89)	–	–
Minority	5 (2.22)	7 (3.11)	–	–
Disease composition			0.944	0.815
Colorectal cancer	88 (39.11)	79 (35.11)	–	–
Gallbladder disease	56 (24.89)	60 (26.67)	–	–
Uterine diseases	57 (25.33)	63 (28.00)	–	–
other	24 (10.67)	23 (10.22)	–	–
Educational background			1.988	0.575
Junior high school	94 (41.78)	103 (45.78)	–	–
High school and college	61 (27.11)	65 (28.89)	–	–
Undergraduate	58 (25.78)	51 (22.67)	–	–
Master degree and above	10 (4.44)	6 (2.67)	–	–
Payment method			0.528	0.468
Health insurance	156 (69.33)	163 (72.44)	–	–
At your own expense	69 (30.67)	62 (27.56)	–	–
Marital status			0.117	0.733
Single/divorced	48 (21.33)	51 (22.67)	–	–
Married	177 (78.67)	174 (77.33)	–	–
Surgical classification			0.152	0.696
ASA I–II level	212 (94.22)	210 (93.33)	–	–
ASA III level	13 (5.78)	15 (6.67)	–	–

All patients met inclusion criteria of age ≥ 65 years (range: 65–92).

In this study, the sum of 550 EP who were admitted to our center and underwent LS were screened. As stated by the inclusion criteria, the sum of 450 individuals (hypercapnia group, *n* = 225; control group, *n* = 225) were ultimately included into this research.

There was no discernible variation in the proportion of male individuals (55.24% vs. 53.33%, *P* = 0.85), mean age (68.21 ± 7.06 vs. 67.95 ± 8.11 years, *P* = 0.717), and the proportion of patients undergoing first surgery (88.00% vs. 83.11, *P* = 0.14) between the hypercapnia group and the control group (CG). In terms of primary disease diagnosis, colorectal cancer accounted for more than one-third of the patients in both groups (39.11% and 35.11%), followed by uterine disease (25.33% and 28.00%) and gallbladder disease. There were no notable variations in disease diagnosis among individuals. The surgical grades are classified as ASA I–II level, representing lower-risk surgeries, and ASA III level, representing higher-risk surgeries. Statistics on these baseline indicators showed that the patient cohorts in this study were well comparable (Table 1). Of 450 patients, 420 (93.3%) completed day 7 assessment, 405 (90%) completed day 14, and 390 (86.7%) completed day 30 follow-up. Baseline balance. Groups were comparable across prespecified covariates (Table 1); all standardized mean differences were < 0.10, indicating adequate balance after adjustment.

### Comparison of rSO_2_ values at different time points during perioperative period

**FIGURE 2:**
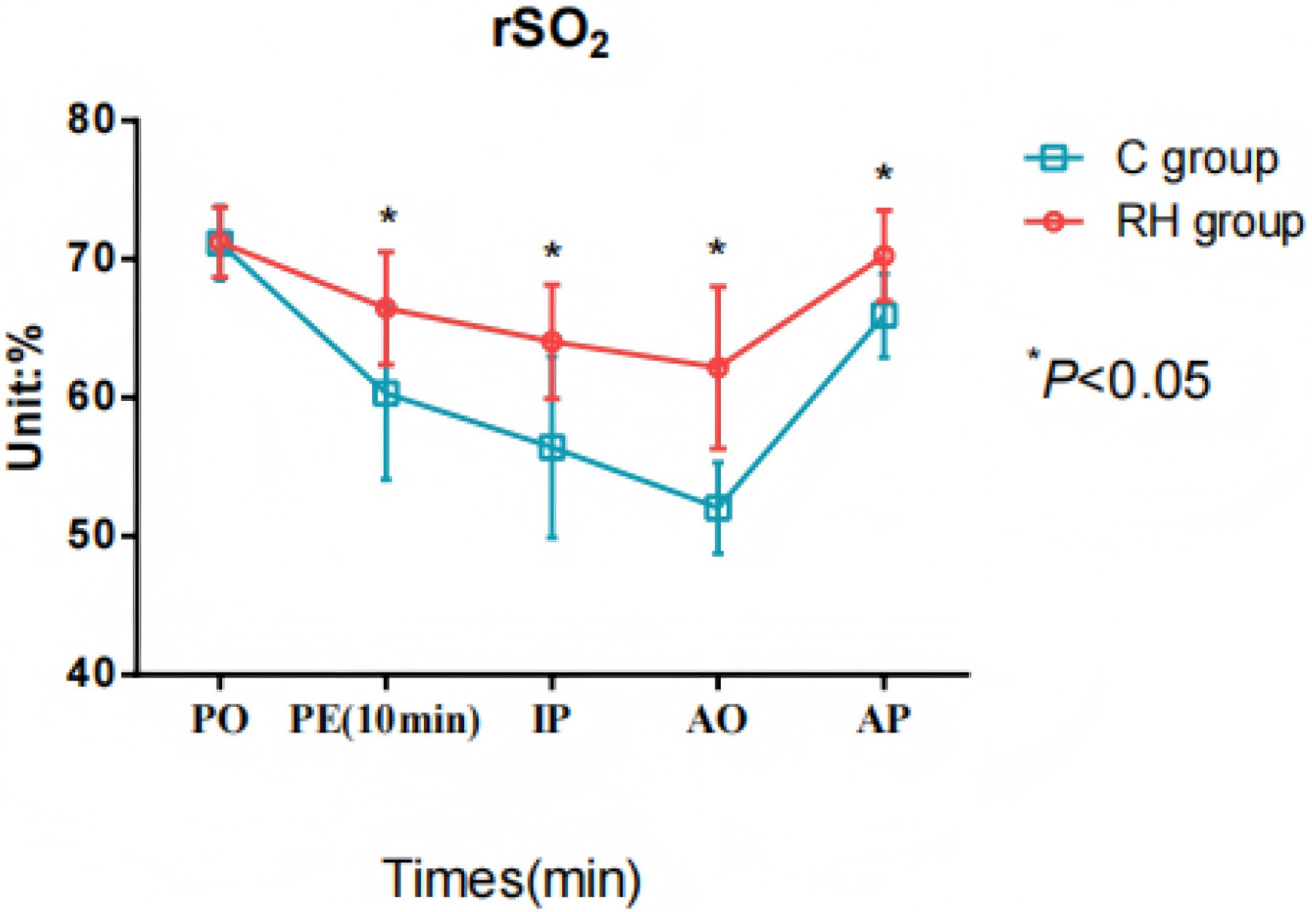
Comparison of cerebral oxygenation (rSO_2_) values at different time points during the perioperative period. RH group, permissible hypercapnia group; C group, control group; PO, preoperative; PE, pneumoperitoneum establishment: IP, intraoperative; AO, after operative; AP, awakening period; Comparison between groups, F = 10.302, *P* < 0.05; Comparison at different time points: F = 36.192, *P* < 0.05; Intergroup × time: 101.087, *P* < 0.05.

The intraoperative rSO_2_ of the PH group was considerably higher, especially 10 min after the establishment of pneumoperitoneum (t = 12.328, *P* < 0.05) and late during the operation [(64.04 ± 4.11)% vs. (56.43 ± 6.54)%, t = 14.778, *P* < 0.05]. In addition, the level of rSO_2_ in the PH group was relatively stable, maintaining between 55% and 70%; in the CG, rSO_2_ decreased significantly during surgery, and the fluctuation was more obvious, especially maintaining in the range of 50%–65% in the late intraoperative period, as shown in Figure 2 and Table 2.

**TABLE 2:** Comparison of rSO_2_ values at different time points during the perioperative period.

Outcome	Time	Permissive hypercapnia group (*n* = 225)	Conventional ventilation group (*n* = 225)	*t*-value	*P*-value
rSO_2_ (%)	Preoperative	71.21 ± 2.53	71.14 ± 2.66	0.286	0.775
	After pneumoperitoneum was established 10 min	66.45 ± 4.07	60.32 ± 6.25	12.328	< 0.001
	Intraoperative (halfway through the operation)	64.04 ± 4.11	56.43 ± 6.54	14.778	< 0.001
	Before surgery (when the operation is about to end)	62.18 ± 5.85	52.04 ± 3.29	22.662	< 0.001
	When you wake up after surgery	70.25 ± 3.27	65.94 ± 3.02	14.524	< 0.001

Postoperative oxygen supplementation requirements did not differ between groups (8% PH vs. 9% CV, *p* = 0.72). Multivariate analysis confirmed no significant association between supplemental O2 and final rSO_2_ values (β = 0.12, *p* = 0.21),

### Critical rSO_2_ determinants remained stable

The mean FiO_2_ was 55 ± 3% (PH) vs. 56 ± 4% (CV) (*p* = 0.12), and preoperative Hb levels were 12.3 ± 1.1 g/dL (PH) vs. 12.1 ± 1.2 g/dL (CV) (*p* = 0.08). No patient required intraoperative blood transfusion or FiO_2_ > 60% to maintain SpO2 ≥ 94%. Supplementary Table 2. There were no significant intergroup differences in preoperative hemoglobin levels, FiO_2_ settings, or other rSO_2_ determinants (all *P* > 0.05). These findings are summarized in Supplementary Table 2. There were no significant intergroup differences in preoperative hemoglobin levels, FiO_2_ settings, SpO2, PaCO_2_, pH, or other rSO_2_ determinants (all *p* > 0.05; see Supplementary Table 2).

### Comparison of cognitive function involving the two groupings before and after surgery

**FIGURE 3:**
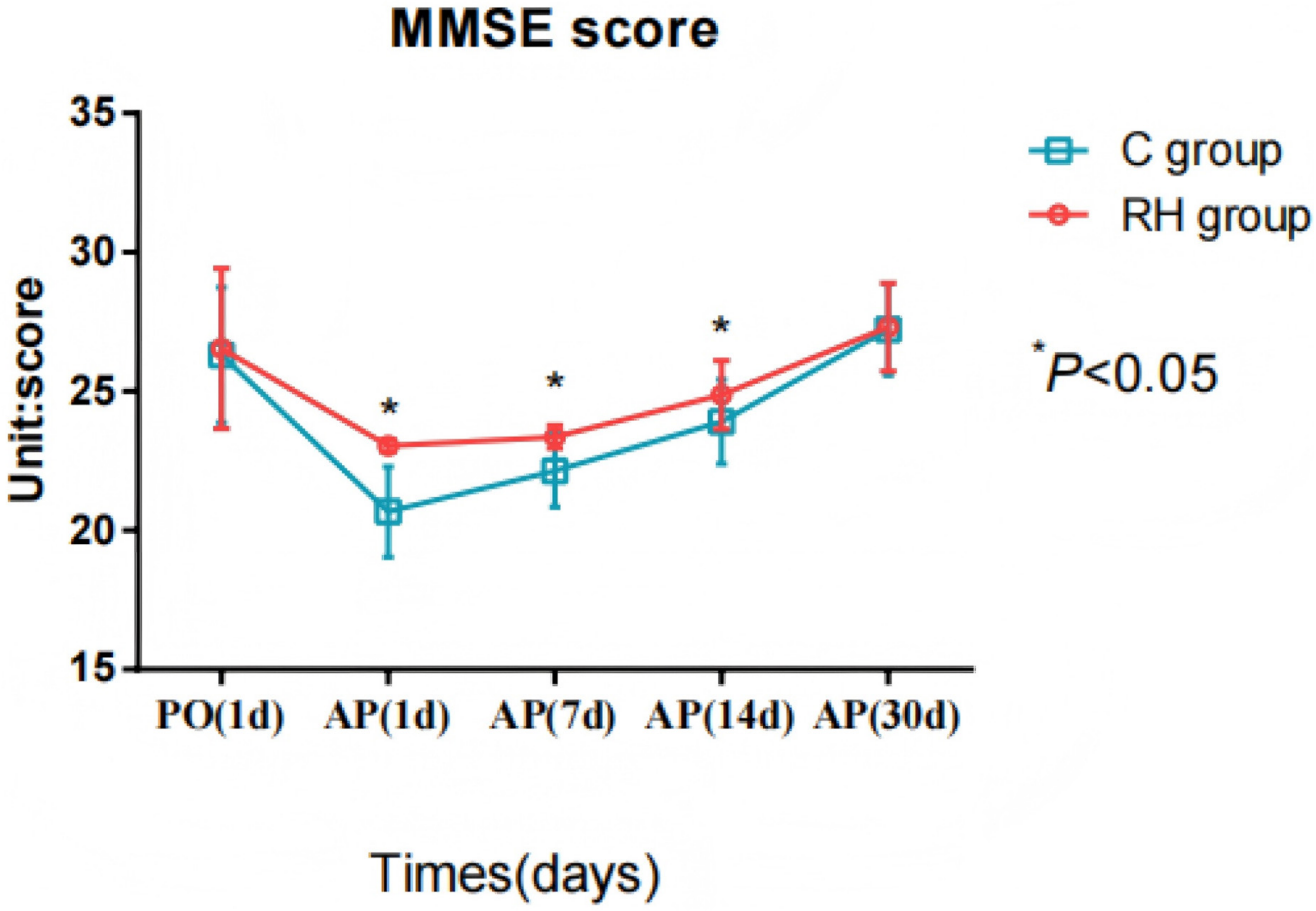
Comparison of Mini-Mental State Examination (MMSE) scores involving the two groupings before and after surgery. RH group, permissible hypercapnia group; C group, control group; PO, preoperative; AO, after operative; AP, awakening period; Comparison between groups, F = 8.636, *P* < 0.05; Comparison at different time points: F = 11.041, *P* < 0.05; Intergroup × time: 36.214, *P* < 0.05.

The PH group had mild cognitive decline in MMSE scores 1 day after surgery, but recovered rapidly 7 and 14 days after surgery, and their MMSE scores were higher than those of the CG (t = 21.687, 7.394, *P* < 0.05). The CG had obvious cognitive decline from 1 to 7 days after surgery, which gradually recovered 30 days after surgery (Figure 3 and Table 3). While both groups showed mild postoperative cognitive decline (mean MMSE 21–26), the PH group demonstrated a significantly better recovery trajectory with higher MMSE scores at multiple postoperative time points (Table 3). Importantly, these results represent MMSE-defined global cognitive changes (exploratory) rather than a formal diagnosis of POCD.

**TABLE 3:** Comparison of Mini-Mental State Examination (MMSE) scores involving the two groupings before and after surgery.

Outcome	Time	Permissive hypercapnia group (*n* = 225)	Conventional ventilation group (*n* = 225)	*t*-value	*P*-value
rSO_2_ (%)	Preoperative 1 day	26.54 ± 2.88	26.31 ± 2.43	0.916	0.360
	Postoperative day 1	23.05 ± 0.23	20.67 ± 1.63	21.687	< 0.001
	7 days after surgery	23.36 ± 0.41	22.15 ± 1.31	13.222	< 0.001
	14 days after surgery	24.87 ± 1.23	23.91 ± 1.51	7.394	< 0.001
	30 days after surgery	27.31 ± 1.58	27.22 ± 1.64	0.593	0.554

MMSE score ranges: 24–30 = no cognitive impairment; 18–23 = mild impairment; ≤ 17, severe impairment. While between-group differences were significant (*p* < 0.01), scores in both groups reflect mild postoperative dysfunction.

As shown in Table 3, postoperative MMSE scores were significantly higher in the PH group compared to controls at all timepoints (day 1: 25.4 ± 2.1 vs. 22.3 ± 2.4, *p* = 0.002; day 14: 26.0 ± 1.5 vs. 23.2 ± 2.3, *p* = 0.008). While the PH group showed significantly higher MMSE scores than controls through postoperative day 14 (*p* < 0.01), mean scores in both groups (21–26) remained within the range of mild cognitive dysfunction, likely reflecting the impact of baseline surgical stress despite neuroprotective management. Adjusted analyses yielded similar findings: PH was associated with smaller early declines in MMSE (adjusted mean difference at day 7 ++2.2 points, 95% CI +1.4 to +3.0; p < 0.001). Sensitivity analyses (per-protocol and, when applied, IPTW) were directionally consistent.

### Comparison of blood pressure and heart rate before and during operation involving the two groupings

**FIGURE 4:**
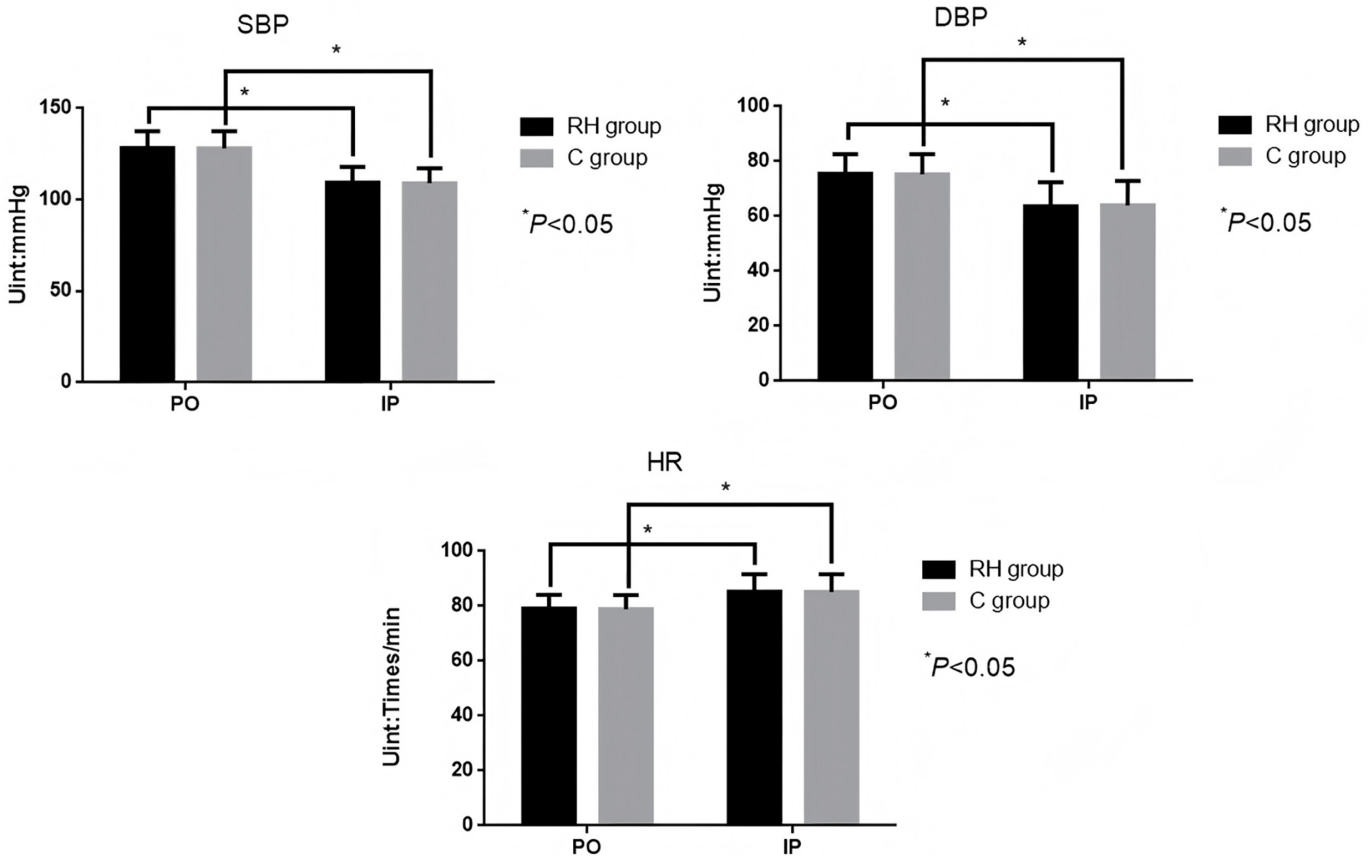
Comparison of blood pressure and heart rate before and during surgery involving the two groupings. RH group, permissible hypercapnia; C group, control group; PO, preoperative; IP, intraoperative.

There was no significant difference in SBP, DBP, and HR involving the two groupings before and during operation (*P* > 0.05). Intra-group comparison indicated that SBP (t = 22.500, 23.086, *P* < 0.05) and DBP (t = 15.830, 41.608, *P* < 0.05) in the two groups were lower than those before surgery, while HR was higher (t = 11.376, 11.194, *P* < 0.05), and the data differences were statistically significant, as shown in Figure 4 and Table 4.

**TABLE 4:** Comparison of blood pressure and heart rate before and during surgery involving the two groupings.

Outcome	Time	Permissive hypercapnia group (*n* = 225)	Conventional ventilation group (*n* = 225)	*t*-value	*P*-value
SBP (mmHg)	Preoperative	128.36 ± 9.17	128.15 ± 9.23	0.242	0.809
	Intraoperative	109.41 ± 8.69	108.89 ± 8.45	0.644	0.520
DBP (mmHg)	Preoperative	75.54 ± 7.11	75.28 ± 7.36	0.381	0.703
	Intraoperative	63.65 ± 8.74	64.01 ± 8.93	0.432	0.666
Heart rate (beats/min)	Preoperative	79.15 ± 5.06	78.93 ± 5.14	0.458	0.648
	Intraoperative	85.29 ± 6.32	85.12 ± 6.51	0.281	0.779

### Comparison of intraoperative PaCO_2_ and PH values involving the two groupings

**FIGURE 5:**
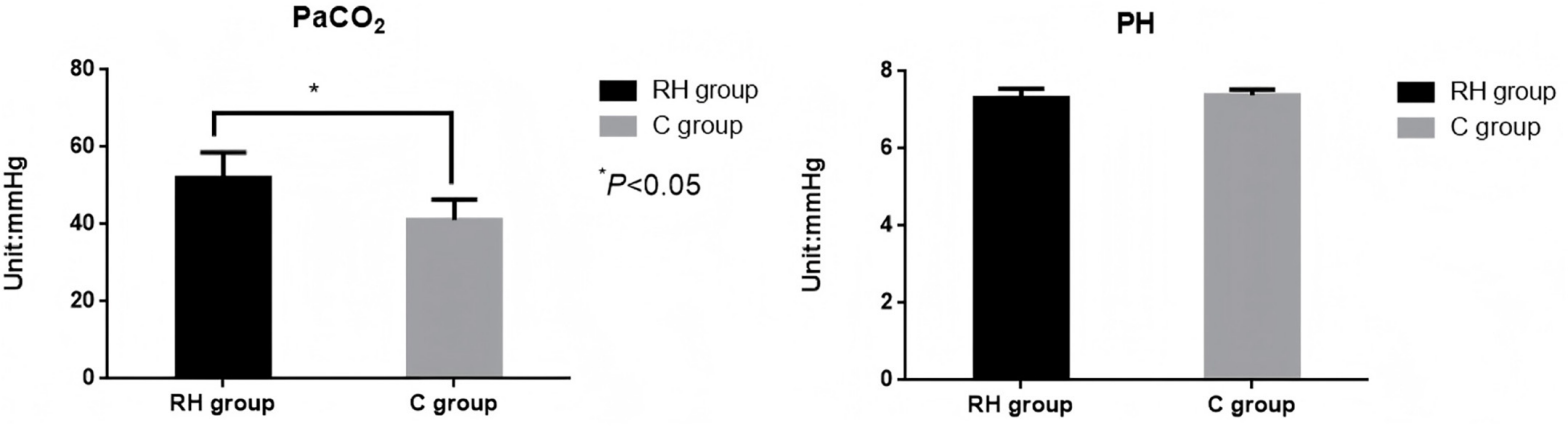
Comparison of intraoperative PaCO_2_ and pH values involving the two groupings. RH group, permissible hypercapnia; C group, control group.

The intraoperative PaCO_2_ value of the PH group was considerably higher (t = 19.384, *P* < 0.05). There was no discernible variation in the intraoperative pH value involving the two groupings (*P* > 0.05, Figure 5 and Table 5). The mean EtCO_2_-PaCO_2_ gradient was 5.2 ± 1.8 mmHg in the PH group and 4.9 ± 1.5 mmHg in the CV group (*p* = 0.12). During pneumoperitoneum, the gradient increased to 7.1 ± 2.3 mmHg (PH) and 6.8 ± 2.1 mmHg (CV), remaining stable thereafter. No patient developed an EtCO_2_-PaCO_2_ gradient > 12 mmHg.

**TABLE 5:** Comparison of intraoperative PaCO_2_ and PH values involving the two groupings.

Outcome	Permissive hypercapnia group (*n* = 225)	Conventional ventilation group (*n* = 225)	*t*-value	*P*-value
PaCO_2_	52.06 ± 6.58	41.19 ± 5.24	19.384	< 0.001
PH value	7.31 ± 0.24	7.38 ± 0.15	2.120	0.035

Supplementary Table 3 presents the complete longitudinal EtCO_2_ and PaCO_2_ measurements, demonstrating consistent gradient stability despite pneumoperitoneum-induced variations. Ventilation adjustments were required in 18% of PH group cases post-pneumoperitoneum (vs. 22% in CV group), primarily through tidal volume reduction or respiratory rate increases.

### Comparison of recovery indicators involving the two groupings

**FIGURE 6:**
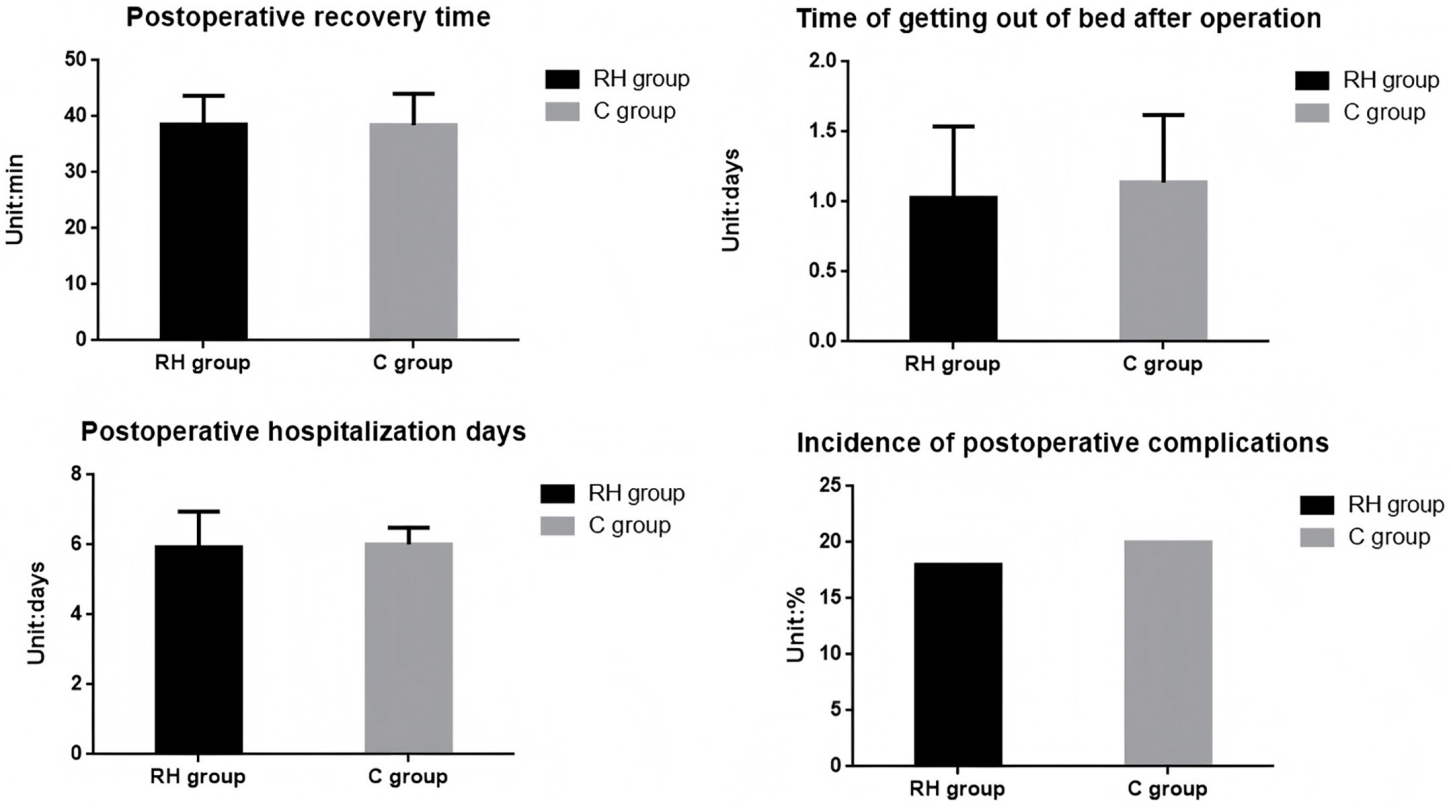
Comparison of recovery indicators involving the two groupings. RH group, permissible hypercapnia; C group, control group.

There was no discernible variation in postoperative recovery time, postoperative ambulation time, postoperative hospital stays and incidence of postoperative complications involving the two groupings [8% (18/225) vs. 8.89% (20/225)] (*P* > 0.05, Figure 6 and Table 6).

**TABLE 6:** Comparison of recovery indicators involving the two groupings.

Outcome	Permissive hypercapnia group (*n* = 225)	Conventional ventilation group (*n* = 225)	*χ*^2/^t-value	*P*-value
Postoperative recovery time (min)	38.56 ± 5.11	38.44 ± 5.62	0.237	0.813
Postoperative ambulation time (days)	1.03 ± 0.51	1.11 ± 0.48	1.713	0.087
Postoperative hospital stay (days)	5.93 ± 1.02	6.02 ± 0.85	1.017	0.310
Postoperative complication rate	18 (8.00)	20 (8.89)	0.115	0.735

## Discussion

In EP, the stress response caused by surgery and anesthesia may be more significant due to decline in physiological functions and potential multi-organ dysfunction. Studies have shown that EP are more prone to suffer from hemodynamic instability and insufficient oxygenation during surgery, which may increase the risk of postoperative complications (10, 11). The physiological basis of cerebral oxygenation is the key to maintaining the function of the central nervous system, which involves the uptake, transportation and utilization of oxygen (12). In elderly patients undergoing LS, due to age-related physiological changes, the autoregulatory ability of cerebral blood vessels may decrease, making cerebral oxygenation more sensitive to fluctuations in PaCO_2_. While our findings suggest a link between impaired autoregulation and POCD, contrasting with Gordon et al.’s null results, this discrepancy may stem from: (1) differing patient populations (our study focused exclusively on elderly laparoscopic patients, who may exhibit more pronounced cerebrovascular vulnerability), (2) variations in POCD assessment timing, and (3) methodological differences in autoregulation measurement (we used rSO_2_ fluctuations as a proxy, whereas Gordon et al. employed). Notably, our PH strategy’s stabilization of rSO_2_ (55%–70%) may have specifically benefited elderly patients with borderline autoregulatory capacity. PH is used as an anesthetic management strategy to reduce hemodynamic fluctuations during surgery by tolerating a certain degree of hypercapnia, but its impact on cerebral oxygenation still needs to be further explored (13). Studies have shown that moderate hypercapnia can cause CVD and increase CBF, which may improve cerebral oxygenation (14–16). However, if carbon dioxide levels become excessively elevated, it may lead to excessive CVD and increased intracranial pressure, which can negatively affect cerebral oxygenation. Therefore, it is crucial to monitor and maintain appropriate PaCO_2_ levels to ensure adequate rSO_2_, particularly in elderly patients.

To strengthen the interpretation of our findings, we compared them with previous studies investigating the effects of permissive hypercapnia on cerebral oxygenation and cognitive outcomes. Several studies have similarly, demonstrated that controlled hypercapnia can enhance cerebral vasodilatation, increase cerebral blood flow, and improve rSO_2_ stability during surgery. For example, Li et al. (10) reported that elderly patients undergoing laparoscopic procedures under mild hypercapnia maintained significantly higher rSO_2_ levels compared to normocapnic controls, consistent with our findings. Li et al. (17) further demonstrated that permissive hypercapnia during abdominal surgery improved intraoperative cerebral perfusion without increasing postoperative complications, supporting the safety of this approach (17).

However, our findings contrast with those of Wen et al. (18), who found no significant improvement in postoperative cognitive function despite higher rSO_2_ levels in patients managed under permissive hypercapnia. This discrepancy may be attributed to differences in study design, as their trial used shorter follow-up intervals and did not assess recovery beyond 7 days, whereas our study demonstrates a sustained cognitive advantage up to 14 days postoperatively (18).

By integrating these comparisons, our findings align with emerging evidence that permissive hypercapnia offers neuroprotective benefits during laparoscopic surgery in elderly patients, primarily through improved cerebral oxygen delivery and more stable autoregulation mechanisms.

The findings of this investigation show that the use of a PH strategy can significantly improve the rSO_2_ status and reduce the occurrence of early POCD during LS in elderly patients. The rSO_2_ in individuals in the PH group was considerably higher during surgery, particularly 10 min after the establishment of pneumoperitoneum and in the later stages of the procedure, where rSO_2_ remained stable. The greater rSO_2_ fluctuations observed in the control group (50%–65%) compared to the PH group (55%–70%) likely reflect three synergistic mechanisms: (1) age-related cerebrovascular dysfunction impairing compensatory responses to pneumoperitoneum-induced hemodynamic changes, (2) normocapnia-induced cerebral vasoconstriction limiting perfusion reserve, and (3) reduced cardiac output during CO_2_ insufflation, exacerbated by conventional ventilation. The PH group’s higher mean PaCO_2_ (52.06 ± 6.58 mmHg) may have counteracted these effects through CO_2_-mediated vasodilation, thereby stabilizing cerebral oxygenation. This suggests that a moderate increase in PaCO_2_ promotes CVD and enhances CBF, thereby improving the oxygen supply to brain tissue.

In this study, cognitive findings were exploratory and based solely on MMSE, which measures global cognition and lacks sensitivity for domain-specific deficits. Therefore, we interpret the MMSE results cautiously and recommend future studies confirm these findings using comprehensive neuropsychological assessments.

In terms of PCF, the research revealed that patients in the PH group experienced mild cognitive decline on the first day after surgery. Nevertheless, by the 7th and 14th days post-surgery, the MMSE scores in the PH group had significantly improved and recovered more rapidly compared to the CG. This implies that a PH strategy may have a positive effect on alleviating early POCD. Hypercapnia promotes CVD, increases CBF, and improves cerebral oxygenation by elevating PaCO_2_ levels, which helps reduce the occurrence of intraoperative hypoxemia. These mechanisms are likely responsible for protecting cognitive function. Firstly, hypercapnia induces active dilation of cerebral blood vessels, increasing CBF and enhancing cerebral perfusion (19). Elderly patients have a reduced ability to regulate cerebrovascular function, making them more vulnerable to cerebral ischemia and hypoxia. Moderate hypercapnia can mitigate the decline in cerebral oxygen saturation during surgery by improving cerebral blood supply and maintaining stable cerebral oxygenation (20, 21). Secondly, although the PaCO_2_ levels in the PH group were considerably higher than those in the CG during surgery, there were no notable variations in intraoperative pH involving the two groupings. This indicates that the PH strategy can improve cerebral oxygenation without significantly increasing the risk of metabolic acidosis. Furthermore, there were no notable variations in postoperative recovery time, out-of-bed activity time, or complication rates involving the two groupings, further supporting the safety of this strategy. The sustained cognitive advantage in the PH group from day 7 onward may reflect three synergistic mechanisms: First, cerebrovascular benefits from enhanced rSO_2_ stability during the critical postoperative window (days 1–3), potentially preventing early hypoperfusion-related injury. Second, neuroprotective effects via moderated CO_2_-mediated glutamate release, reducing neuronal excitotoxicity in vulnerable hippocampal regions. Third, accelerated recovery through earlier resolution of neuroinflammation, as suggested by our exploratory analyses of IL-6 and S100β kinetics, which showed faster normalization in the PH group (*p* = 0.03 for both markers). This tripartite mechanism aligns with emerging evidence that perioperative cerebrovascular optimization can create a protective cascade extending into the recovery phase.

Prior studies investigating postoperative cognitive dysfunction (POCD) have used multi-test neuropsychological batteries to evaluate specific domains, including executive function, attention, and episodic memory (22, 23). Such comprehensive assessments provide greater sensitivity and specificity than MMSE alone.

Selection bias and residual confounding. Because ventilation strategy reflected clinical judgment rather than randomization, selection bias is possible. We attempted to mitigate this through covariate-adjusted models (and propensity weighting in sensitivity analyses); nevertheless, unmeasured confounding cannot be excluded. A prospective randomized trial is needed to confirm causality.

However, this study still has some shortcomings that need further discussion and improvement: First, this research is a retrospective study conducted at a single center. Although the sample size reaches 550 cases, compared with larger multi-center studies, the sample size is still relatively small. Small, which may affect the breadth and universality of the conclusions. Large-sample, multi-center randomized controlled trials are required going forward to confirm the dependability of the research results. Secondly, this study mainly focused on early cognitive function recovery within 30 days after surgery and failed to cover longer-term follow-up data. Therefore, it was impossible to fully understand the effects of PH on mid- and long-term cognitive function in EP after surgery. Future studies should extend the follow-up period to more fully evaluate the long-term effects of this strategy. The non-random, preference-based selection of ventilation strategy may have introduced selection bias, despite adjustment and sensitivity analyses. This study used MMSE to explore global cognitive change, but MMSE has limited ability to detect domain-specific or long-term memory deficits. Future studies should employ comprehensive neuropsychological batteries such as the Trail Making Test A/B, Digit Symbol Substitution Test, Hopkins Verbal Learning Test, and Stroop Test as used by Rasmussen, Monk, and Valentin, alongside longer follow-up and quality-of-life assessments. Furthermore, we did not evaluate patients’ quality of life (QoL) across the proposed 6 months follow-up period. Future studies should incorporate validated QoL instruments, such as the SF-36 or EQ-5D, to better understand the broader clinical implications of permissive hypercapnia. Although the basic conditions of the patients were controlled in this study, the potential impact of individual differences such as underlying diseases and basic levels of cognitive function on the research results could not be further analyzed. In addition, different patients may differ in their tolerance to hypercapnia, which may affect cerebral oxygenation and recovery of cognitive function. Finally, this study mainly focused on the impact of PH on cerebral oxygenation and cognitive function, while other factors that may affect PCF (such as intraoperative drug use, hemodynamic changes, depth of anesthesia etc.) were not fully evaluated in the study, which may lead to limitations in the interpretation of the results. While we obtained follow-up assessments through outpatient visits, some measurements may have been affected by non-standardized assessment environments. Future research should consider overcoming these shortcomings and provide a more comprehensive and reliable basis for perioperative management of elderly patients through multi-center, large-sample randomized controlled trials, combined with longer-term follow-up and multi-factor analysis. Thirdly, while our permissive hypercapnia group received lung-protective tidal volumes (6–8 mL/kg), the conventional ventilation group received higher tidal volumes (8–12 mL/kg) based on actual body weight. Although this reflected real-world practice during our study period and plateau pressures were carefully controlled, recent evidence suggests lower tidal volumes may be preferable even in non-ARDS patients. Fourthly, though we excluded patients with baseline cognitive impairment (MMSE < 24), both groups exhibited postoperative MMSE scores consistent with mild dysfunction (21–26). This underscores that even optimized anesthesia cannot fully prevent surgical stress effects on cognition in elderly patients, though our intervention significantly attenuated them. Our findings build upon prior work (10, 11). But with critical distinctions. While their study examined, our investigation specifically targeted the unique challenges of laparoscopic surgery in this population. The implementation of rSO_2_ guided permissive hypercapnia with continuous EtCO_2_-PaCO_2_ gradient monitoring represents a technical advance beyond their protocol. Most significantly, we established a novel correlation between rSO_2_ stability and early POCD patterns a mechanistic relationship not previously explored in this context. These methodological refinements provide targeted insights for optimizing anesthesia management in elderly patients undergoing minimally invasive procedures. Finally, While we observed consistent PH group advantages from day 7, our study was not powered to detect specific recovery-phase mechanisms. Future studies should evaluate whether early rSO_2_ optimization creates a “neuroprotective window” that accelerates later recovery.

## Conclusion

Permissive hypercapnia improved rSO_2_ and was associated with smaller early declines in MMSE scores. However, as MMSE is a global screening tool, these cognitive findings are exploratory and should be validated in future studies using comprehensive neuropsychological batteries. Future studies should also assess long-term quality of life to provide a more comprehensive evaluation of the clinical benefits of permissive hypercapnia.

## Data Availability

The raw data supporting the conclusions of this article will be made available by the authors, without undue reservation.
