# Supplementary material for: Effects of permissive hypercapnia on intraoperative cerebral oxygenation and early postoperative cognitive function in elderly patients undergoing laparoscopic surgery
Source: Front Med (Lausanne). 2025 Oct 9;12:1575412. doi: 10.3389/fmed.2025.1575412 (PMC12546227; doi:10.3389/fmed.2025.1575412)
Supplement: Supplementary file 1 [file Data_Sheet_1.docx]

**Table S1:** Temporal Changes in Ventilation Protocols

| **Year** | **Control Group VT** | **PH Group VT** | **PEEP (cmH₂O)** |
| --- | --- | --- | --- |
| 2019-2021 | 8-12 mL/kg | 6-8 mL/kg | 5-8 |
| 2022-2024 | ≤8 mL/kg | 6-8 mL/kg | 5-8 |

**Table S2:** Postoperative Oxygen Supplementation, Final rSO₂ Values, and Critical Determinants Between Groups

| **Variable** | **PH Group (n = 225)** | **CV Group (n = 225)** | **p-value** |
| --- | --- | --- | --- |
| **Patients receiving supplemental O₂** | 18 / 225 (8%) | 20 / 225 (9%) | 0.72 |
| **Mean rSO₂ (with O₂)** | 68.2 ± 3.1 | 65.1 ± 2.8 | 0.58 |
| **Mean rSO₂ (room air)** | 67.9 ± 3.4 | 64.8 ± 3.0 | 0.61 |
| **FiO₂ (%)** | 55 ± 3 | 56 ± 4 | 0.12 |
| **Preoperative Hb (g/dL)** | 12.3 ± 1.1 | 12.1 ± 1.2 | 0.08 |
| **SpO₂ (%)** | 97 ± 2 | 96 ± 3 | 0.21 |
| **PaCO₂ (mmHg)** | 52.1 ± 6.6 | 39.8 ± 5.5 | <0.001 |
| **pH** | 7.35 ± 0.05 | 7.37 ± 0.04 | 0.09 |

**Table S3:** Intraoperative EtCO₂, PaCO₂, and Ventilation Management

| **Timepoint** | **PH Group (n=225)** | **CV Group (n=225)** | **p-value** | **Ventilation Adjustments Triggered** |
| --- | --- | --- | --- | --- |
|  | EtCO₂ \| PaCO₂ (mmHg) | EtCO₂ \| PaCO₂ (mmHg) |  |  |
| Baseline | 38.2±3.1 \| 43.1±2.8 | 37.9±2.9 \| 42.8±2.6 | 0.45 | None |
| Post-intubation | 49.6±4.2 \| 54.8±3.9 | 38.1±3.3 \| 43.2±2.9 | <0.001 | PH: RR ↑ in 12% |
| Post-pneumoperitoneum | 58.3±5.1 \| 65.4±4.8 | 44.7±3.8 \| 49.6±3.5 | <0.001 | PH: VT ↓ in 18%; CV: RR ↑ in 22% |
| Intraoperative (hourly) | 56.2±4.7 \| 61.5±4.3 | 42.5±3.5 \| 47.3±3.2 | <0.001 | PH: VT ↓ in 9%; CV: RR ↑ in 15% |
| Pre-extubation | 50.1±4.0 \| 55.3±3.7 | 39.8±3.1 \| 44.5±2.8 | <0.001 | None |
| Gradient (ΔEtCO₂-PaCO₂) | 5.2±1.8 | 4.9±1.5 | 0.12 |  |

- PH Group: Tidal volume (VT) decreased if PaCO₂ >65 mmHg or EtCO₂ >65 mmHg
- CV Group: Respiratory rate (RR) increased if PaCO₂ >45 mmHg or EtCO₂ >45 mmHg
